# Ultrasound-assisted leaching of vanadium from fly ash using lemon juice organic acids

**DOI:** 10.1039/c9ra09325g

**Published:** 2020-01-10

**Authors:** G. Rahimi, S. O. Rastegar, F. Rahmani Chianeh, T. Gu

**Affiliations:** Chemical Engineering Group, Department of Engineering, University of Kurdistan Sanandaj Iran so.rastegar@uok.ac.ir +98 8733664343; Department of Chemical and Biomolecular Engineering, Ohio University Athens OH 45701 USA

## Abstract

In this work, vanadium (V) was selectively extracted from fuel-oil fly ash using a leaching process utilizing organic acids extracted from lemon juice with assistance from ultrasound and H_2_O_2_. Response Surface Methodology (RSM) was used to optimize the main operating factors. The V recovery was 88.7% at the optimal conditions: 27.9% (v/v) lemon juice, 10% (v/v) hydrogen peroxide (H_2_O_2_), solid/liquid (S/L) ratio 0.01% (w/v), ultrasound power 159 W at 20 kHz in 2 h, and initial temperature of 35 °C. The effect of time on the V recovery was examined. The maximum recovery was 100% after 3 h. Furthermore, the individual effects of ultrasound and H_2_O_2_ on V recovery were studied, and the results showed that without H_2_O_2_ and ultrasound, the V recovery decreased greatly, indicating that both factors were essential in the leaching process. According to the modified shrinking core model, test results indicated that mass diffusion was the controlling step of the overall reaction kinetics. The activation energy of the leaching reaction in the temperature range 25 to 65 °C was found to be 17.1 kJ mol^−1^.

## Abstract 2

In this work, vanadium (V) was selectively extracted from fuel-oil fly ash using a leaching process utilizing organic acids extracted from lemon juice with assistance from ultrasound and H_2_O_2_.

## Graphical abstract

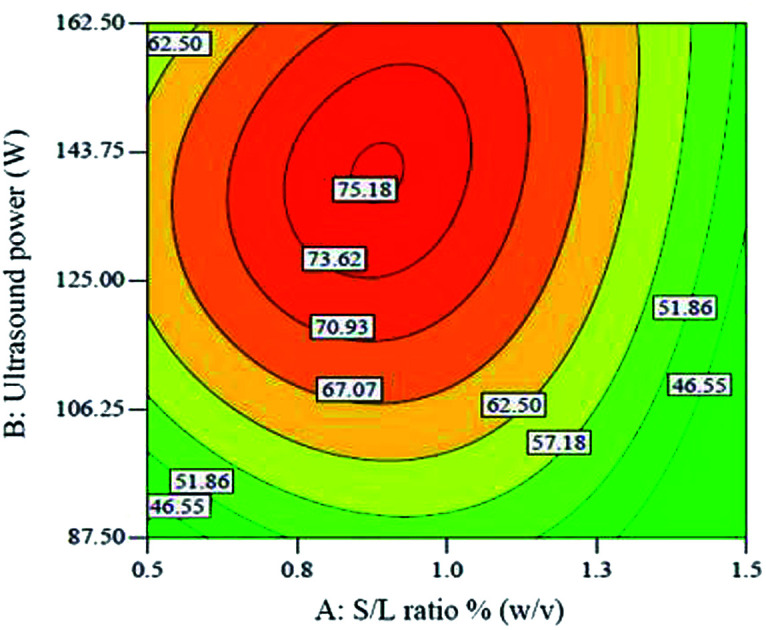

## 1. Introduction

Every year large volumes of bottom and fly ashes are generated around the world by burning fuel oil in thermal power plants.^1^ These ashes contain valuable metals such as vanadium (V), nickel (Ni), copper (Cu), zinc (Zn), cobalt (Co), *etc.*, which have many industrial applications.^1,2^ On the other hand, if there is no treatment of fly ashes, they would create many environmental problems, including soil, water and air pollution.^2–4^ Therefore, the extraction of metals from produced fly ashes is very desirable from both environmental and economic aspects.^1^

Different chemical and physical processes including pyrometallurgy and hydrometallurgy were used for the extraction of valuable metals from ores and wastes. However, they consume too much energy or create environmental problems.^5,6^ Nowadays, bioleaching is an attractive alternative for metal extraction. Bioleaching relies on microorganisms such as *Acidithiobacillus thiooxidans*, *Acidithiobacillus ferrooxidans* and *Leptospirillum ferrooxidans*, *Aspergillus niger*.^7–9^ The metal extraction mechanisms are based on the production of leaching agents such as ferric ion, sulfuric acid, and different organic acids including citric acid, maleic acid and ascorbic acid are reported in the literature.^8,10,11^ These organic acids dissolve metals by the displacement of metal ions from the solid matrix with hydrogen ions or by the formation of soluble metal complexes and chelates.^10^ Although this method is safe and environmentally friendly, its use for industrial and very toxic feeds is limited. Furthermore, the slow kinetics due to slow microbial growth and nutrient diffusion limitation are bottlenecks in bioleaching.^12^

An alternative way to get organic acids is to use organic acids from juices of fruits such as lemon, lime, *etc.*, which are rich in citric acid, ascorbic acid and malic acid. These biogenic acids are very effective in the extraction of metals.^13,14^ Using acids extracted from agricultural products are not only environmentally friendly but also good at resolving slow kinetics of bioleaching by microorganisms.^15–17^ Moreover, using lemon juice as a renewable source of organic acids, the cost of leaching wastewater treatment is eliminated.

To assist the effect of acid in the leaching process, different methods including ultrasound, H_2_O_2_ and microwave have been used.^18,19^ Ultrasound relies on the cavitation of microbubbles which have a strong effect on a solid surface. It can increase the kinetics and recovery of metal leaching processes. In an ultrasound-assisted bioleaching process for the recovery of Zn, Cu, and Co from black shale, one group of researchers achieved optimal leaching efficiencies of 92% Cu and 87% Zn and 71% Co, respectively, at a sonication time of 7 min with 15 d of pre-growth and 36 d of bioleaching.^20^ In the bioleaching of printed circuit boards (PCBs), Huang *et al.* reported that using 300 W ultrasound waves, 93.8% Cu was recovered compared to 90.7% without ultrasound.^21^ In the bioleaching of Ni from Sukinda laterite, ultrasound reduced the leaching time from 20 d to 14 d.^22^ Ultrasound also increased the removal efficiency of Ni from lateritic nickel ore considerably.^23^

There are several published reports about the extraction of valuable metals from power plant ashes using bioleaching in the literature.^1,2,7^ Leaching using a fruit juice has not been reported before. This work used organic acids from inexpensive lemon juice to extract V in the fly ash from a fuel-oil power plant. Ultrasound and H_2_O_2_ were used to assist the leaching process. Response Surface Methodology (RSM) was applied to optimize the operating factors of the process. The kinetic and thermodynamic parameters of the process were also investigated.

## 2. Materials and methods

### 2.1. Preparation of the fly ash

**Fig. 1:**
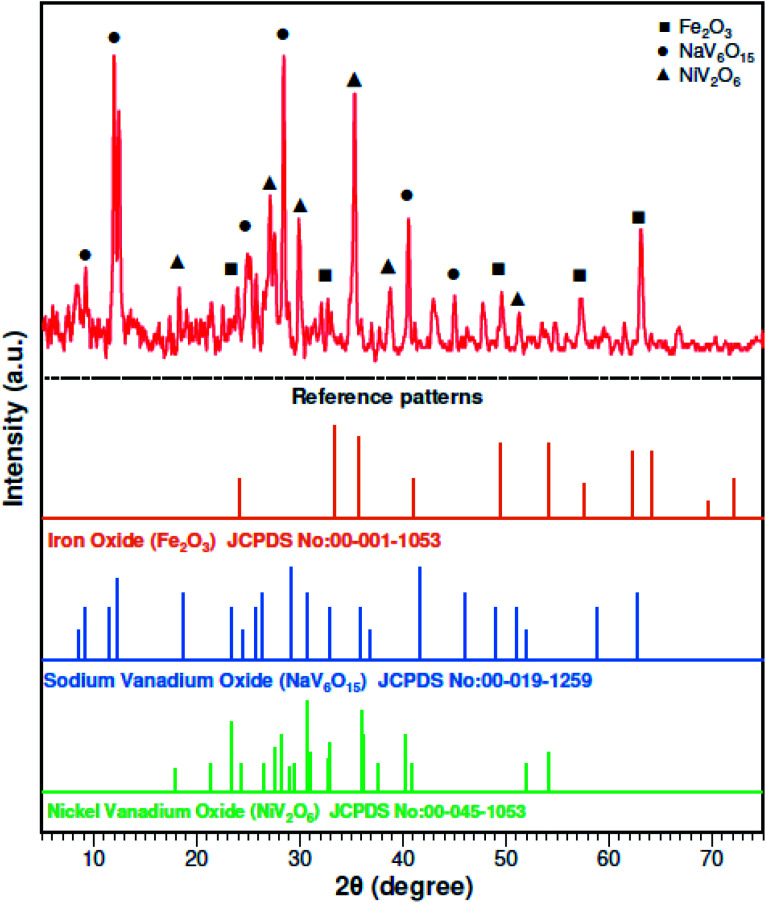
XRD patterns of the raw power plant ash.

A fuel-oil fly ash sample, labeled as power plant residue (PPR), was collected from the Neka Thermal Power Plant, Mazandaran, Iran. It was crushed and screened through 75 μm sieves. The raw PPR was washed with hexane and acetone (1 : 1 mass ratio), and the slurry was mixed with a magnetic stirrer for 120 min at 50 °C. Then, a fine powder was obtained by filtering the slurry through a 0.40 μm Whatman filter paper. The powder was dried at 70 °C for 0.5 h before use. Aqua regia (hydrochloric acid and nitric acid with a molar ratio of 3 : 1) was prepared for the complete digestion of PPR in 24 h at 50 °C. Inductively coupled plasma optical emission spectrometry (ICP-OES) (Vista Pro, Varian, Inc., California, USA) was used to analyze chemical elements in the solid samples. The chemical elements in the raw PPR are shown in Table 1. Fig. 1 shows the X-ray diffraction (XRD) (X'Pert MPD, Philips, Netherlands) patterns and it was determined that the initial PPR sample was mostly Fe_2_O_3_, NaV_6_O_15_ and NiV_2_O_6_.

**Table 1:** Elemental composition of the raw power plant ash

Element	Al	Cu	Fe	Ni	V
Concentration (ppm by volume)	90.45	5.25	1059	1346	4665

### 2.2. Chemicals

H_2_O_2_ (30% by volume), hexane, acetone, hydrochloric acid (HCl), nitric acid (HNO_3_), sodium carbonate (Na_2_CO_3_), calcium chloride (CaCl_2_), and ammonium chloride (NH_4_Cl), all in analytical grade, were provided by Merck (Germany). Distilled water was used for preparing all aqueous solutions.

### 2.3. Preparation of lemon juice

Lemon was procured from a local market in Sanandaj, Iran. A kitchen juicer was used to extract lemon juice. In order to remove the pulp, the raw juice was centrifuged for 15 min at a speed of 3000 rpm. High performance liquid chromatography (HPLC) (LC20AD, Shimadzu, Japan) was used to measure the concentrations of organic acids in the lemon juice. The concentration of citric acid in lemon juice was 90 mg g^−1^ (juice), whereas the concentration of malic acid and ascorbic acid were 0.86 mg g^−1^ and 1.24 mg g^−1^, respectively.

### 2.4. Analytical methods

A portable pH and Eh meter (Metrohm, Switzerland) was used to measure pH and Eh. To determine the concentrations of the metal ions in the leaching solutions, ICP-OES was used. The elemental composition in the V precipitate was determined using X-ray fluorescence (XRF) (PW1410, Philips, Netherlands). Surface characteristics of the PPR ash before and after the leaching process were determined using a field emission scanning electron microscope (FE-SEM) (TSCAN, Czech Republic).

### 2.5. Experimental design

Design Expert 7.0.0 software was adopted to optimize the leaching parameters. RSM was applied to design the tests. Response Surface Methodology (RSM) is a statistical method used to improve, analyze, and optimize processes.^2^ Central Composite Design (CCD) was applied for studying the effects of four main factors, namely ultrasound power, lemon juice percentage, H_2_O_2_ percentage, and solid/liquid (S/L) ratio on V recovery. Table 2 shows the coded values of the variables. A CCD analysis using the four factors resulted in 30 runs which are listed in Table 3. In this study, the percent of V extracted was considered the experimental response. Analysis of variance (ANOVA) was used to determine the significant models and parameters. The behavior of the system was interpreted using the empirical polynomial model described by the following equation:^2^1
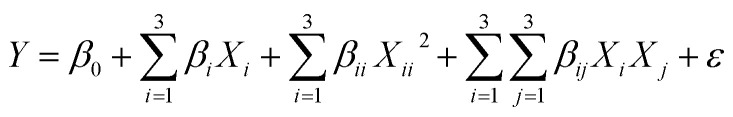
where *Y* is the model response, *β*_0_ the model constant. *β*_*i*_, *β*_*ii*_ and *β*_*ij*_ are linear, quadratic and interaction coefficients, respectively. *X*_*i*_ represents independent factors, and *ε* is error. Finally, the obtained models were validated by comparing the experimental results carried out at optimal conditions with the model predicted results.

**Table 2:** Experimental factors at different levels used for the ultrasound-assisted leaching experiment at 35 °C for 2 h

Factor	Code	Unit	Low axial (−2)	Low factorial (−1)	Centre point (0)	High factorial (+1)	High axial (+2)
S/L ratio	*A*	%	0.01	0.5	1.0	1.5	2
Ultrasound power	*B*	W	50	88	125	163	200
Lemon juice conc.	*C*	%	0	20	40	60	80
H_2_O_2_ conc.	*D*	%	0	5	10	15	20

**Table 3:** Experimental plan based on CCD and the results of V extraction

Run	H_2_O_2_ conc. (%)	Lemon juice conc. (%)	Ultrasound power (W)	S/L ratio%	V recovery (%)
1	15	60	163	1.5	49.0
2	10	40	125	1.0	54.3
3	10	0	125	1.0	13.8
4	0	40	125	1.0	8.2
5	5	20	163	1.5	43.8
6	5	60	163	0.5	49.6
7	15	20	163	1.5	48.9
8	15	20	88	1.5	50.4
9	10	40	125	1.0	45.4
10	5	60	163	1.5	40.9
11	15	20	88	0.5	46.4
12	15	60	163	0.5	53.1
13	15	20	163	0.5	62.3
14	10	40	200	1.0	54.8
15	5	20	88	1.5	39.4
16	5	60	88	1.5	37.9
17	10	40	125	1.0	41.6
18	15	60	88	0.5	44.4
19	10	80	125	1.0	42.0
20	10	40	125	1.0	43.0
21	10	40	125	1.0	46.5
22	10	40	125	2.0	40.8
23	10	40	50	1.0	39.9
24	20	40	125	1.0	58.3
25	10	40	125	0.01	79.6
26	5	20	163	0.5	62.6
27	5	60	88	0.5	43.9
28	10	40	125	1.0	48.5
29	5	20	88	0.5	47.0
30	15	60	88	1.5	32.8

### 2.6. Leaching experiment

According to the test conditions in Table 2, certain amounts of the PPR ash, lemon juice and H_2_O_2_ were put into a 150 mL glass beaker. An ultrasonic probe (UF55/UN55, Memmert, Germany) was inserted in the solution during leaching. The ultrasound generator was set at varying power in the range from 50 W to 400 W at fixed 20 kHz frequency. After the leaching was terminated, the spent solid was removed from the liquor using centrifugation for 15 min at a speed of 3000 rpm. The ICP-OES analysis was used to determine the concentrations of metals in the liquor. The V recovery was calculated using the following equation:2
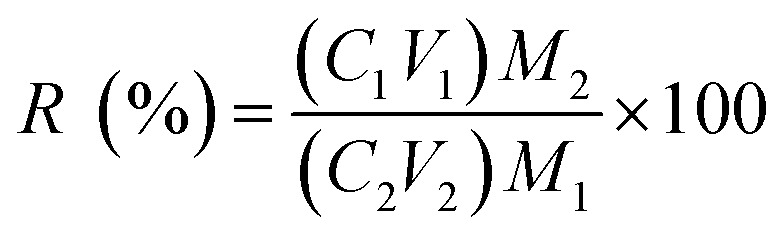
where *R* is leaching efficiency or recovery of the metal (%), *C*_1_ and *C*_2_ are the metal concentrations (ppm by volume) in aqua regia and the liquor after leaching, respectively, *M*_1_ and *V*_1_ are the solid mass and aqua regia volume, respectively, and *M*_2_ and *V*_2_ are the solid mass and liquor volume in the bioacid leaching liquor, respectively. All these leaching tests, except in the thermodynamic study, lasted 2 h with an initial temperature of 35 °C. Due to ultrasound energy input, the leaching solution temperature typically rose by 20 °C after 2 h. The variations of Eh and pH during the leaching process were monitored.

### 2.7. V precipitation from liquor

In order to precipitate V selectively from the leaching liquor after the leaching process, the impurities in the liquor were removed first by increasing the liquor pH to 9–10 using 1 M sodium carbonate (Na_2_CO_3_), and then adding 0.1% (w/v) of CaCl_2_. Afterwards, the solution was stirred vigorously for 30 min at 80 °C before the solution was filtered using a 0.42 μm Whatman filter paper to precipitate other metal ions from the solution. In the next step, NH_4_Cl was added to the solution to precipitate V in the liquor as ammonium metavanadate (NH_4_VO_3_) while the solution was stirred for 1 h. The obtained NH_4_VO_3_ precipitate was separated from the solution using a 0.42 μm Whatman filter paper and then calcinated at 550 °C for 4 h in a muffle furnace to obtain V_2_O_5_.^24^

## 3. Results and discussion

### 3.1. Statistical analysis

The ANOVA outcome of the data and the results are shown in Table 4. A reduced cubic model was used for the V extraction. The correlation between V recovery and the four defined factors is shown as follows:

3 V recovery (%) = 43.65 − 9.71*A* + 4.16*B* + 7.06*C* + 12.54*D* − 1.60*AB* + 1.11*AD* + 0.78*BD* + 4.57*A*^2^ + 1.35*B*^2^ − 3.52*C*^2^ + 1.95*ABC* − 1.01*ACD* + 1.48*BCD* − 11.28*A*^2^*D* + 5.70*AB*^2^ − 10.27*B*^2^*C*

where *A*, *B*, *C*, and *D* are S/L ratio, ultrasound power, lemon juice% (v/v), and H_2_O_2_% (v/v), respectively. The relatively high R-squared and adjusted R-squared values in Table 4 indicated that the model was a good fit with experimental results, thus validating the suitability of the model. Moreover, results showed that at a 95% confidence level, the calculated *p*-value of the model was less than 0.05 (Table 4), indicating that the model was statistically significant. The adequate precision, which indicated the signal to noise (S/N) ratio, was 15.4, much greater than the commonly accepted threshold of 4.

**Table 4:** ANOVA of RSM outcome^a^

Source	Sum of squares	*df*	Mean square	*F*-Value	*p*-Value	
Model	4560	16	285	8.7	0.0002	Significant
*A* – S/L ratio	754	1	754	23.2	0.0003	
*B* – ultrasound power	414	1	415	12.8	0.0034	
*C* – lemon juice	399	1	399	12.3	0.0039	
*D* – H_2_O_2_	1258	1	1258	38.7	<0.0001	
*AB*	41.1	1	41.1	1.3	0.2806	
*AD*	19.6	1	19.6	0.6	0.4505	
*BD*	9.8	1	9.8	0.3	0.5919	
*A* ^2^	585	1	585	18.0	0.0010	
*B* ^2^	51.3	1	51.3	1.6	0.2309	
*C* ^2^	346.0	1	346	10.6	0.0061	
*ABC*	61.0	1	61.0	1.9	0.1936	
*ACD*	16.3	1	16.3	0.5	0.4913	
*BCD*	34.9	1	34.9	1.1	0.3184	
*A* ^2^ *D*	678	1	678	20.9	0.0005	
*AB* ^2^	173	1	173	5.3	0.0379	
*B* ^2^ *C*	563	1	563	17.3	0.0011	
Residual	422	13	32.5			
Lack of fit	392	9	43.5	5.7	0.0537	Not significant

a *R* ^2^ = 0.91; adj. *R*^2^ = 0.81; C.V. (%) = 12.5; adeq. precision = 15.4.

### 3.2. Contour plots

**Fig. 2:**
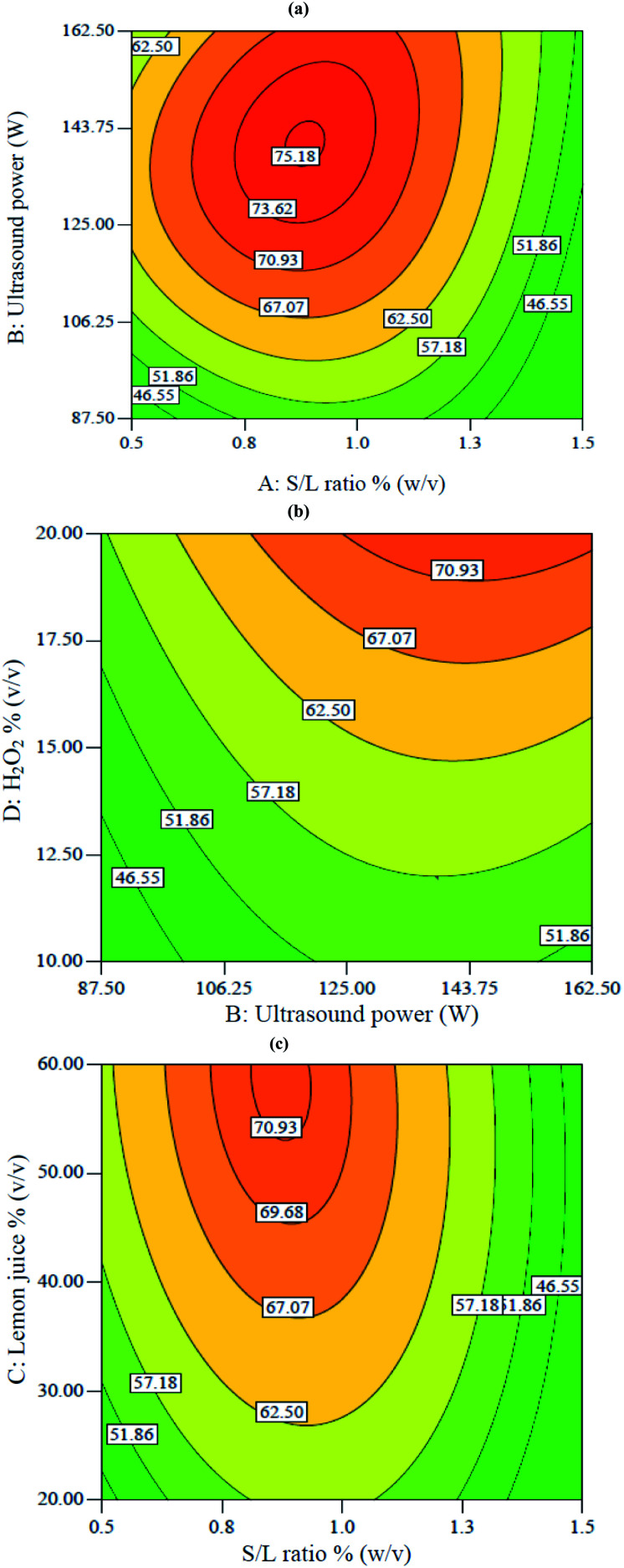
Contour plots of the interactive effects on V extraction: (a) interaction between S/L ratio with power at constant 60% lemon juice and 20% H_2_O_2_, (b) interaction between power and H_2_O_2_ concentration at constant 0.75% S/L ratio and 50.3% lemon juice, (c) interaction between S/L ratio and lemon juice at constant 117 W power and 20% H_2_O_2_.

Fig. 2 shows two-dimensional graphs for V extraction. These graphs show the interactions between different parameters on the V recovery. Fig. 2a shows the interaction between S/L ratio and ultrasound power at constant 20% H_2_O_2_ and 60% lemon juice. According to Fig. 2a, a maximum of 75.1% V extraction was obtained at 0.9% S/L ratio and 140 W ultrasound power. Decreasing S/L ratio from 1.5 to 0.9% and increasing ultrasound power from 87 to 143 W led to increased V recovery. Decreasing S/L ratio resulted in decreased viscosity of the mixture and diffusion mass transfer resistance, thus promoting acid penetration into the solid particles. This led to increased V recovery.^18^ The positive effect of increased ultrasound power was due to enhanced cavitation, leading to the breaking of chemical bonds.^25^

**Fig. 3:**
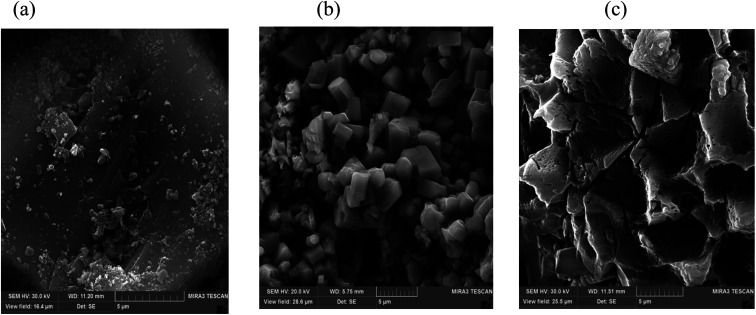
FESEM images of (a) raw ash, (b) leaching without ultrasound, and (c) leaching with ultrasound.

Fig. 2b shows the interaction between H_2_O_2_ and ultrasound power at constant 50.3% lemon juice and 0.75% S/L ratio. According to Fig. 3b, the V extraction was enhanced when ultrasound power and H_2_O_2_ concentration increased from 87 W to 162 W and 10% to 20%, respectively. Increasing both ultrasound power and H_2_O_2_ percentage led to increased solution potential (Eh) which resulted in enhanced leaching efficiency. The positive effect of ultrasound in the presence of H_2_O_2_ was due to the breaking of the chemical bond between vanadium and oxygen.^26,27^

The following equation shows the reaction between NiV_2_O_6_ and citric acid in the presence of H_2_O_2_:4
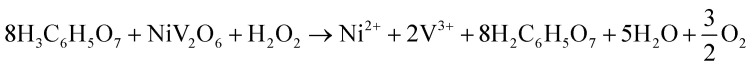


Fig. 2c shows the interaction between lemon juice concentration and S/L ratio when ultrasound power and H_2_O_2_ percentage were fixed at 117 W and 20%, respectively. It shows that the V recovery increased from 57% to 71% when lemon juice concentration increased from 20% to 60% at constant 0.9% S/L ratio. The concentration of lemon juice (containing three acids, namely citric acid, malic acid and ascorbic acid) increased the availability of H^+^ ions and acted as the oxidant during the leaching process. The dissociation of H^+^ from citric acid was shown in eqn (5)–(7):^28^

5 H_3_Cit(aq) → H_2_Cit^−^ + H^+^ p*K*_a_1__ = 3.15

6 H_2_Cit^−^ → HCit^2−^ + H^+^ p*K*_a_2__ = 4.77

7 HCit^2−^ → Cit^3−^ + H^+^ p*K*_a_3__ = 6.4

Similarly, the dissociation of H^+^ from malic acid are expressed in eqn (8) and (9):^28^

8 H_2_C_4_H_4_O_5_ → HC_4_H_4_O_5_^−^ + H^+^ p*K*_a_1__ = 3.4

9 HC_4_H_4_O_5_^−^ → C_4_H_4_O_5_^2−^ + H^+^ p*K*_a_2__ = 5.04

Another positive effect of the lemon juice was due to the carboxyl and hydroxyl groups of in the organic acids in the lemon juice. After losing a proton, they became chelating agents, which formed a complex with a metal as shown in eqn (10), where M is a metal in the solid matrix and R–(COOH)_*m*_ organic acid.^28,29^

10 Ash–M + R–(COOH)_*m*_ → Ash + R–M(COOH)_*m*_

### 3.3. Process optimization and model validation

The goal of optimization was to achieve the highest V extraction. At the optimum conditions, the maximum V extraction was 88.7%. The optimal conditions were 28.0% lemon juice, 10.0% H_2_O_2_ at 0.01% S/L ratio, and ultrasound power of 159 W. In order to verify the suitability of the model, a test was conducted at the optimal conditions predicted by the model. Results indicated that the experimental optimal V recovery (88.7%) was not far from the model prediction (79.7%). The experimental result fell within the 95% confidence interval (C.I.) of the model value.

### 3.4. The roles of H_2_O_2_ and ultrasound

The control tests to understand the V recovery change by skipping a leaching agent (ultrasound and H_2_O_2_) were performed. Results indicated that in the presence of ultrasound and H_2_O_2_ under optimal conditions, the V recovery was 88.7%. However, without ultrasound the metal extraction decreased to 37.5%. It is known that ultrasound waves can increase metal recovery by creating cavitation and bubbles in the liquid. The bubbles explode with increasing pressure. As a result of the energy released by the bursting of bubbles at the particle surface, the metal recovery is increased.^30,31^ Moreover, Run 4 in Table 4 was done without H_2_O_2_ and result showed the V recovery decreased greatly to 8.2%, indicating that H_2_O_2_ was also essential in achieving good V recovery.

### 3.5. Particle morphology analysis

**Fig. 4:**
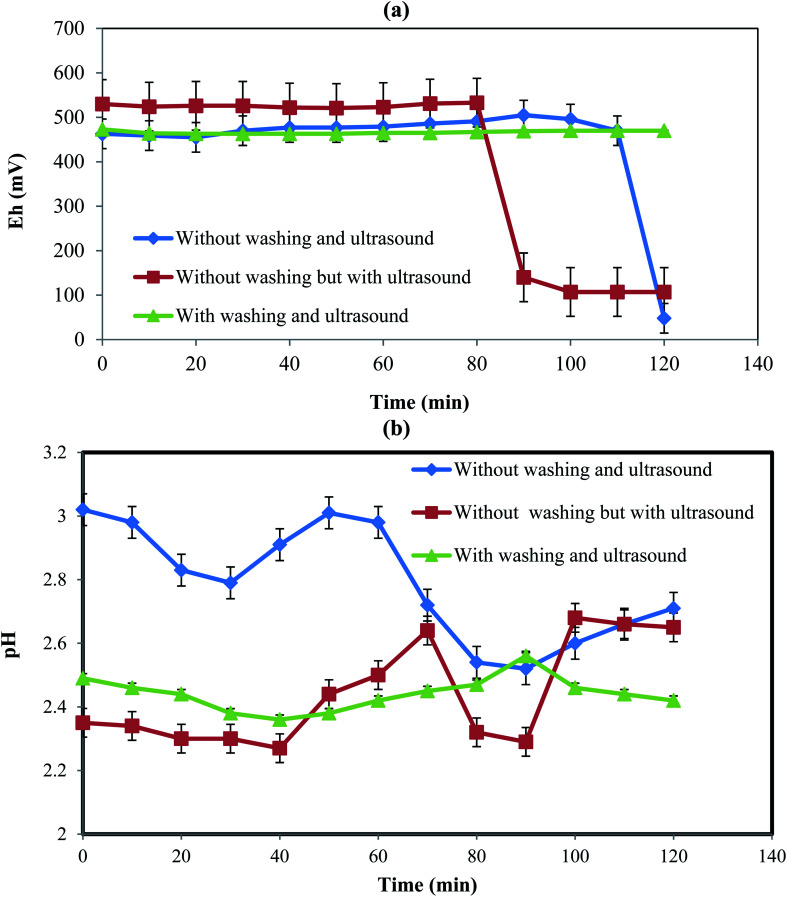
Eh and pH variations with time for different conditions.

In order to show the effects of ultrasound and lemon juice acids, FESEM was used to analyze the particle surfaces of the raw PPR ash (Fig. 3a), the residue after leaching without ultrasound (Fig. 3b) and the residue after leaching with ultrasound (Fig. 3c). By comparing Fig. 4a with Fig. 3(b and c), it can be seen that leaching reduced particle sizes.

A comparison of Fig. 3b with Fig. 3c also suggests that ultrasound eroded the particle surface considerably which resulted in increased penetration of lemon juice acids into internal particle surfaces.

### 3.6. Eh and pH variations

Fig. 4 shows the variations of Eh and pH at three different conditions including (a) without the initial PPR ash washing in the absence of ultrasound, (b) without the initial PPR ash washing in the presence of ultrasound, and (c) with initial washing and in the presence of ultrasound.

#### 3.6.1. Eh variation

Fig. 4a shows the changes of Eh with time at different conditions tested above. In the first case without the initial PPR ash washing in the absence of ultrasound, the Eh of the PPR suspension remained almost constant at about 464 mV until 110 min due to the presence of an impermeable oily layer on the dirty particles. After 110 min, Eh decreased from 467 mV to 48 mV due to the elimination of the oily layer as the diffusion barrier and the H_2_O_2_ reacted with the internal surfaces of the particles.

However, in the second case without the initial PPR ash washing in the presence of ultrasound, due to the existence of the impermeable oily layer around the solid particles, the hydroxyl radicals of H_2_O_2_ in the solution were not consumed and then the amount of Eh remained almost constant at about 533 mV for the first 80 min. The initial potential for a constant Eh range was numerically larger than in the previous case (without ultrasound). This was due to the production of hydroxyl radicals induced by ultrasound.^32^ After 80 min, due to the breakdown of the diffusion layer and the consumption of hydroxyl radicals, Eh decreased to 140 mV. In this case, the breaking of the oily layer occurred 30 min earlier than the previous case because ultrasound accelerated the breaking.

In the third case with washing and ultrasound, the initial washing removed the oily layer which resulted in consumption of hydroxyl radicals at the beginning of the process and at the same time ultrasound produced hydroxyl radicals. Thus, the consumption and production of hydroxyl radical were simultaneous from the beginning, leading to almost constant Eh without a sharp drop.

#### 3.6.2. pH variation

Fig. 4b indicates the variations of pH during time in different cases. In the first case without the initial PPR ash washing in the absence of ultrasound, pH decreased till 30 min, which was due to the release of H^+^ from acids in the lemon juice according eqn (5)–(9). Then, until about 60 min, the produced H^+^ ions were consumed to remove the oily layer, causing pH to increase. From 60 to 100 min, pH decreased again due to the degradation of the oily layer as well as the release of H^+^ from more acid molecules.

In the second case without the initial PPR ash washing in the presence of ultrasound, the pH level was lower than in the previous case, which was the result of ultrasound dissolution citric, malic and ascorbic acids that led to pH reduction early on. The pH had fewer fluctuations than in the first case, which was due to the ultrasound action that destroyed the oily layer, as well as its acceleration of the production and consumption of H^+^.

In the third case with washing and ultrasound, the pH fluctuations are much less pronounced than the two previous cases. This was the result of the fast destruction of the oily layer and the fact that the production and consumption of H^+^ were almost simultaneous.

### 3.7. Kinetic study

Experimental results showed that with increasing time, the V recovery at the optimal conditions predicted from RSM increased and the maximum was 100% after 3 h.

**Fig. 5:**
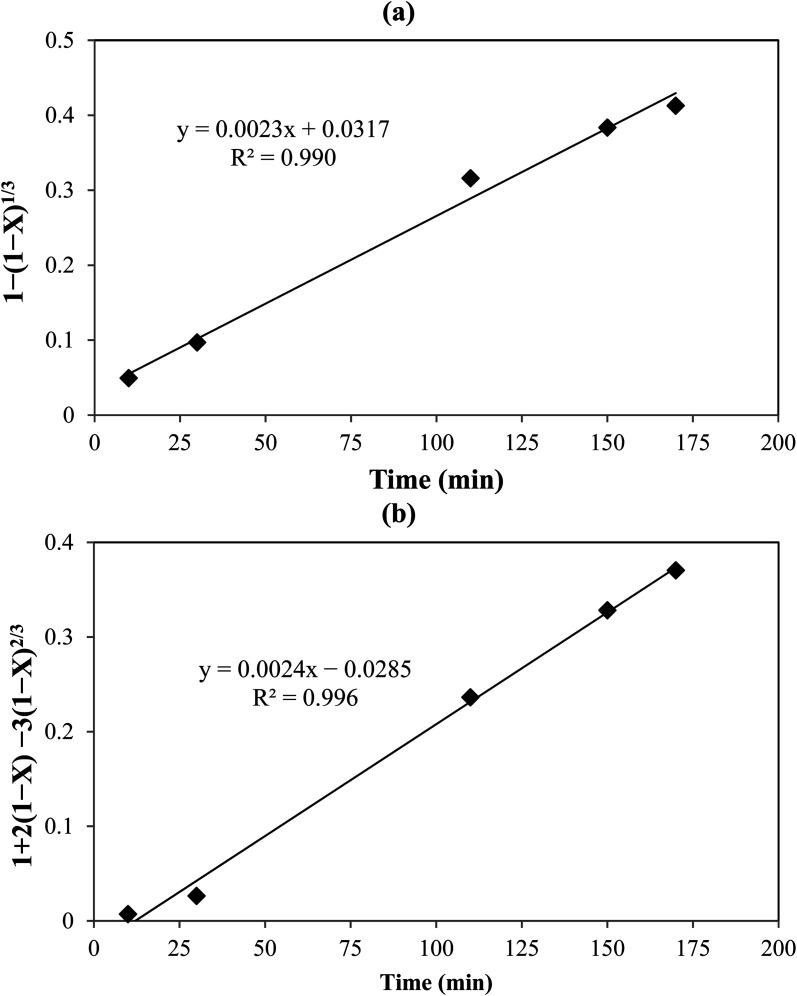
Shrinking core model: (a) with chemical reaction control, and (b) with diffusion control.

The kinetic parameters of the process were analyzed using the shrinking core model which represents the speed of reactions in penetrating the solid network and conducting a chemical reaction in the particle surface layer. Based on the model, the diffusion-control and the reaction-control scenarios are presented in eqn (11) and (12), respectively:^33,34^11
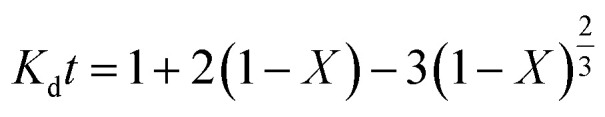
12
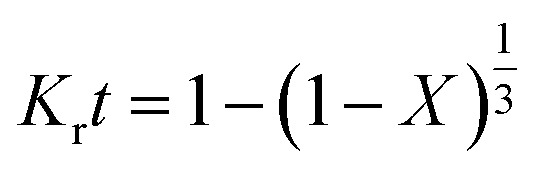
where *X* is leaching efficiency (%), *K*_r_ reaction-controlled rate constant (min^−1^), *K*_d_ diffusion-controlled reaction rate constant (min^−1^), and *t* leaching time (min). Fig. 5 shows the plots of eqn (10) and (11). The obtained *R*^2^ values indicated that the data were fitted with the diffusion-controlled model slightly better than the reaction-controlled model.

### 3.8. Thermodynamic modeling

The rate equation for the reaction in eqn (4) can be expressed as follow:

13 *r* = *kk*_1_

where

14 *k*_1_ = [H_3_C_6_H_5_O_7_]^*a*^[H_2_O_2_]^*b*^[Ni^+^]^*c*^[V^5+^]^*d*^[H_2_C_6_H_5_O_7_]^*e*^[O_2_]^*f*^[H_2_O]^*g*^

in which *a*–*g* values are reaction orders. Using the definition of *k* based on the Arrhenius equation (eqn (15)),15
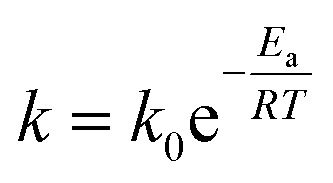


Eqn (13) can be rearranged to give eqn (16):16
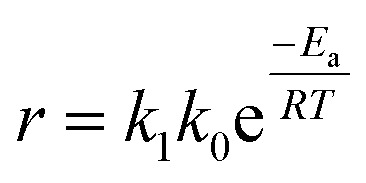
where *R* is universal gas constant (8.314 J mol^−1^ K^−1^), *E*_a_ activation energy of reaction and *T* absolute temperature. The linearized equation of eqn (16) is eqn (17)17
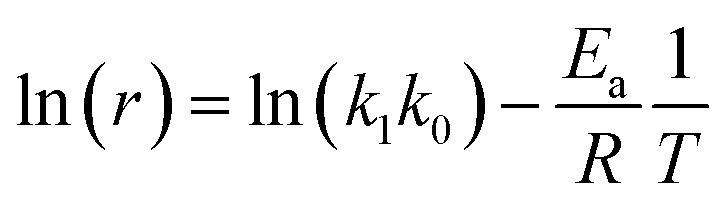


**Fig. 6:**
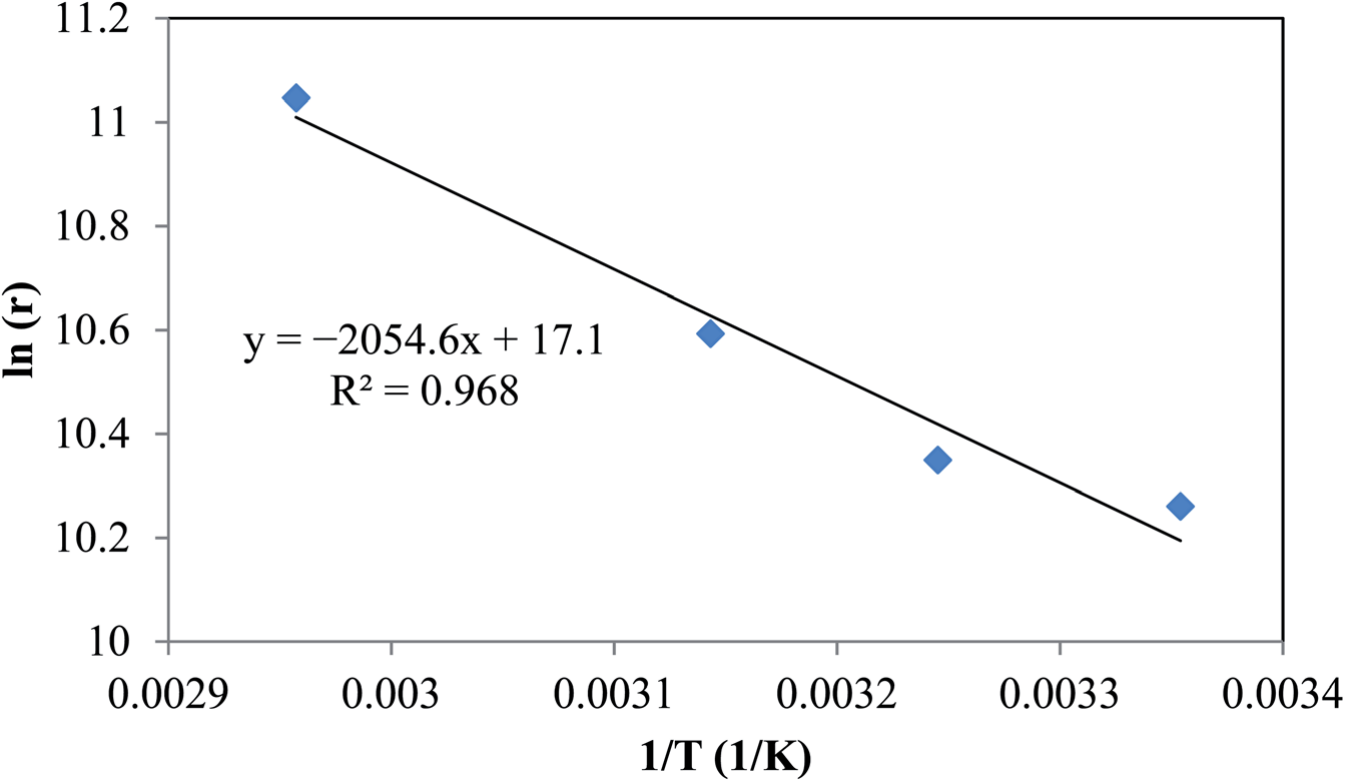
Linear regression of logarithm of reaction rate and 1/*T*.

By plotting ln(*r*) *vs.* 1/*T* (Fig. 6), the activation energy was estimated to be 17.1 kJ mol^−1^ which is less than 25 kJ mol^−1^. This suggests diffusion control in the leaching process.

### 3.9. Separation and purification of V

After the bioacid leaching, many ions such as Fe, Ni, and Cu existed in the solution. These impurities had to be removed before V precipitation. It was found the adding CaCl_2_ to the solution at pH 9–10 could remove other metal ions with a high efficiency. V ion in this pH range is soluble. NH_4_Cl was added to precipitate V as shown in eqn (18). Finally, ammonium metavanadate (NH_4_VO_3_) was formed with a low solubility that led to a precipitate as the product.^2^

18 NaVO_3_ + NH_4_Cl → NH_4_VO_3_↓ + NaCl

**Fig. 7:**
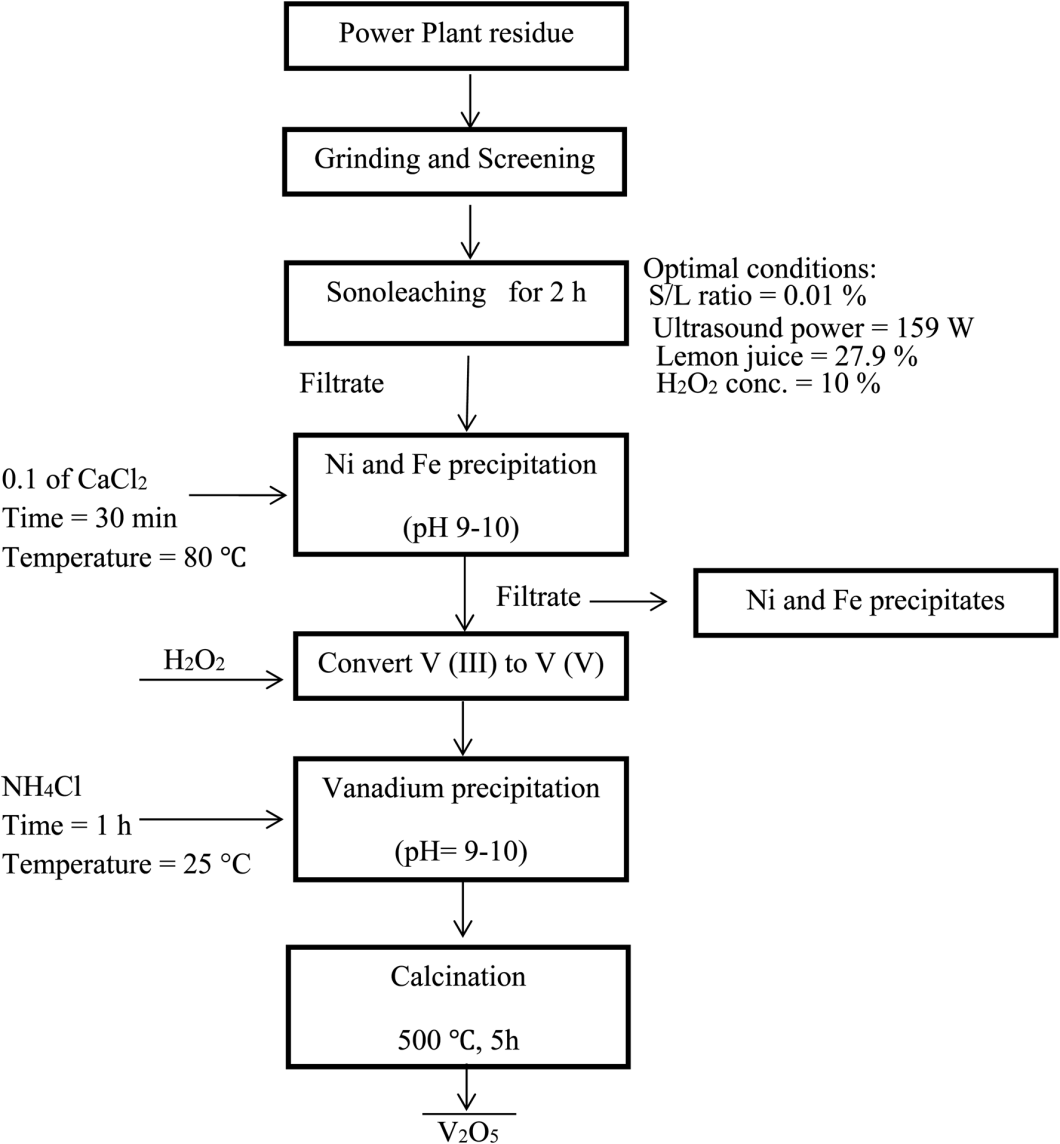
Flowchart of an integrated process for leaching of PPR ash and subsequent selective precipitation of V.

Fig. 7 shows a summary of the overall leaching-purification process. XRF analysis was applied to determine the composition of the precipitate and the results in Table 5 indicate that V was selectively precipitated with a high purity.

**Table 5:** Composition of the final V precipitate

Na_2_O (%)	TiO_2_ (%)	MnO (%)	Fe_2_O_3_ (%)	Ni (ppm)	V (%)
0.885	0.033	0.017	0.146	55	3.15

## 4. Conclusion

The leaching of V from a PPR ash was carried out using bioacids from lemon juice with assistance of ultrasound and H_2_O_2_. The recovery of V was optimized using RSM. The optimal leaching conditions were found to be 27.9% lemon juice, 10% H_2_O_2_, S/L ratio of 0.01% and ultrasound power of 159 W which resulted in 88.7% V extraction in 2 h with initial temperature 35 °C experimentally. Additionally, the effects of skipping one of the two assisting leaching agents (H_2_O_2_ and ultrasound) were assessed. It was found that both were essential for good V recovery. The reaction kinetics analysis results showed that the diffusion-control model was favored over the overall reaction-control model. The activation energy of the reaction was found to be 17.1 kJ mol^−1^ in the temperature range 25 to 65 °C. Finally, V was purified from the leaching solution by precipitation as ammonium metavanadate with a high purity.

## Conflicts of interest

There are no conflicts to declare.

## Supplementary Material

## References

[cit1] Rasoulnia P., Mousavi S. M., Rastegar S. O., Azargoshasb H. (2016). Fungal leaching of valuable metals from a power plant residual ash using *Penicillium simplicissimum*: evaluation of thermal pretreatment and different bioleaching methods. Waste Manag..

[cit2] Rastegar S. O., Mousavi S. M., Shojaosadati S. A. (2015). Bioleaching of an oil-fired residual: process optimization and nanostructure NaV_6_O_15_ synthesis from the bioleachate. RSC Adv..

[cit3] Zhou H., Bhattarai R., Li Y., Li S., Fan Y. (2019). Utilization of coal fly and bottom ash pellet for phosphorus adsorption: sustainable management and evaluation. Resour., Conserv. Recycl..

[cit4] Yue Y., Liu Z., Liu Z., Zhang J., Lu M., Zhou J., Qian G. (2019). Rapid evaluation of leaching potential of heavy metals from municipal solid waste incineration fly ash. J. Environ. Manage..

[cit5] Jadhav U. U., Hocheng H. (2012). A review of recovery of metals from industrial waste. Journal of Achievements in Materials and Manufacturing Engineering.

[cit6] Xin B., Jiang W., Aslam H., Zhang K., Liu C., Wang R., Wang Y. (2012). Bioleaching of zinc and manganese from spent Zn–Mn batteries and mechanism exploration. Bioresour. Technol..

[cit7] Dong Y. B., Yue L., LIN H., LIU C. J. (2019). Improving vanadium extraction from stone coal via combination of blank roasting and bioleaching by ARTP-mutated *Bacillus mucilaginosus*. Trans. Nonferrous Met. Soc. China.

[cit8] Bahaloo-Horeh N., Mousavi S. M., Baniasadi M. (2018). Use of adapted metal tolerant *Aspergillus niger* to enhance bioleaching efficiency of valuable metals from spent lithium-ion mobile phone batteries. J. Cleaner Prod..

[cit9] Lu Y., Zheng G., Zhou W., Wang J., Zhou L. (2019). Bioleaching conditioning increased the bioavailability of polycyclic aromatic hydrocarbons to promote their removal during co-composting of industrial and municipal sewage sludges. Sci. Total Environ..

[cit10] Amiri F., Yaghmaei S., Mousavi S. M. (2011). Bioleaching of tungsten-rich spent hydrocracking catalyst using *Penicillium simplicissimum*. Bioresour. Technol..

[cit11] Kursunoglu S., Ichlas Z. T., Kaya M. (2018). Dissolution of lateritic nickel ore using ascorbic acid as synergistic reagent in sulphuric acid solution. Trans. Nonferrous Met. Soc. China.

[cit12] Wei X., Liu D., Li W., Liao L., Wang Z., Huang W., Huang W. (2018). Biochar addition for accelerating bioleaching of heavy metals from swine manure and reserving the nutrients. Sci. Total Environ..

[cit13] Campo G., Berregi I., Caracena R., Ignacio Santos J. (2006). Quantitative analysis of malic and citric acids in fruit juices using proton nuclear magnetic resonance spectroscopy. Anal. Chim. Acta.

[cit14] Karadenuz F. (2004). Main Organic Acid Distribution of Authentic Citrus Juices in Turkey. Turk. J. Agric. For..

[cit15] Gu T., Rastegar S. O., Mousavi S. M., Li M., Zhou M., Zhang X. (2018). Advances in bioleaching for recovery of metals and bioremediation of fuel ash and sewage sludge. Bioresour. Technol..

[cit16] Pathak A., Morrison L., Healy M. G. (2017). Catalytic potential of selected metal ions for bioleaching, and potential techno-economic and environmental issues: a critical review. Bioresour. Technol..

[cit17] Asghari I., Mousavi S. M. (2014). Effects of key parameters in recycling of metals from petroleum refinery waste catalysts in bioleaching process. Rev. Environ. Sci. Bio/Technol..

[cit18] Jiang F., Chen Y., Ju S., Zhu Q., Zhang L., Peng J., Wang X., Miller J. D. (2018). Ultrasound-assisted leaching of cobalt and lithium from spent lithium-ion batteries. Ultrason. Sonochem..

[cit19] Petrović Sanja J., Bogdanović Grozdanka D., Antonijević Milan M. (2018). Leaching of chalcopyrite with hydrogen peroxide in hydrochloric acid solution. Trans. Nonferrous Met. Soc. China.

[cit20] Anjum F., Bhatti H. N., Ghauri M. A. (2010). Enhanced bioleaching of metals from black shale using ultrasonics. Hydrometallurgy.

[cit21] Huang Z., Xie F. C., Ma Y. (2011). Ultrasonic recovery of copper and iron through the simultaneous utilization of Printed Circuit Boards (PCB) spent acid etching solution and PCB waste sludge. J. Hazard. Mater..

[cit22] Sukla L. B., Swamy K. M., Narayana K. L., Kar R. N., Panchanadikar V. V. (1995). Bioleaching of Sukinda laterite using ultrasonics. Hydrometallurgy.

[cit23] Kar R. N., Sukla L. B., Swamy K. M., Panchanadikar V. V., Narayana K. L. (1996). Bioleaching of lateritic nickel ore by ultrasound. Metall. Mater. Trans. B.

[cit24] Navarro R., Guzman J., Saucedo I., Revilla J., Guibal E. (2007). Vanadium recovery from oil fly ash by leaching, precipitation and solvent extraction processes. Waste Manag..

[cit25] Entezari M. H., Kruus P. (1996). Effect of frequency on sonochemical reactions II. temperature and intensity effects. Ultrason. Sonochem..

[cit26] Li L., Ge J., Chen R., Wu F., Chen S., Zhang X. (2010). Environmental friendly leaching reagent for cobalt and lithium recovery from spent lithium-ion batteries. Waste Manag..

[cit27] Saeki S., Lee J., Zhang Q., Saito F. (2004). Co-grinding LiCoO_2_ with PVC and water leaching of metal chlorides formed in ground product. Int. J. Miner. Process..

[cit28] Golmohammadzadeh R., Faraji F., Rashchi F. (2018). Recovery of lithium and cobalt from spent lithium ion batteries (LIBs) using organic acids as leaching reagents: a review. Resour., Conserv. Recycl..

[cit29] Suanon F., Sun Q., Dimon B., Mama D., Yu C. P. (2016). Heavy metal removal from sludge with organic chelators: comparative study of N,N-bis(carboxymethyl) glutamic acid and citric acid. Environ. Manag..

[cit30] Marafi M., Stanislaus A. (201150). Waste catalyst utilization: extraction of valuable metals from spent hydroprocessing catalysts by ultrasonic-assisted leaching with acids. Ind. Eng. Chem. Res..

[cit31] Li L., Qu W., Zhang X., Lu J., Chen R., Wu F., Amine K. (2015). Succinic acid-based leaching system: a sustainable process for recovery of valuable metals from spent Li-ion batteries. J. Power Sources.

[cit32] Vays S., Ting Y. P. (2017). A review of the application of ultrasound in bioleaching and insights from sonication in (bio) chemical processes. Resources.

[cit33] Levenspiel O. , Chemical Reaction Engineering, Wiley, New York, 3rd edn, 1998

[cit34] Sadeghi N., Moghaddam J., Ojaghi Ilkhchi M. (2017). Kinetics of zinc sulfide concentrate direct leaching in pilot plant scale and development of semi-empirical model. Trans. Nonferrous Met. Soc. China.

