# Correction: Ultrasound-assisted leaching of vanadium from fly ash using lemon juice organic acids

**DOI:** 10.1039/d0ra90109a

**Published:** 2020-10-19

**Authors:** G. Rahimi, S. O. Rastegar, F. Rahmani, T. Gu

**Affiliations:** Chemical Engineering Group, Department of Engineering, University of Kurdistan Sanandaj Iran so.rastegar@uok.ac.ir +98 8733664343; Department of Chemical and Biomolecular Engineering, Ohio University Athens OH 45701 USA

## Abstract

Correction for ‘Ultrasound-assisted leaching of vanadium from fly ash using lemon juice organic acids’ by G. Rahimi *et al.*, *RSC Adv.*, 2020, **10**, 1685–1696, DOI: 10.1039/C9RA09352G.

The authors regret that the name of one of the authors (Farhad Rahmani) was shown incorrectly in the original article. The corrected author list is as shown above.

The Royal Society of Chemistry apologises for these errors and any consequent inconvenience to authors and readers.

## Supplementary Material

